# Naked eye detection of multiple tumor-related mRNAs from patients with photonic-crystal micropattern supported dual-modal upconversion bioprobes†
†Electronic supplementary information (ESI) available: Experimental details and supplementary results. See DOI: 10.1039/c6sc03401b



**DOI:** 10.1039/c6sc03401b

**Published:** 2016-08-19

**Authors:** Xiaoxia Hu, Yingqian Wang, Haoyang Liu, Jie Wang, Yaning Tan, Fubing Wang, Quan Yuan, Weihong Tan

**Affiliations:** a Key Laboratory of Analytical Chemistry for Biology and Medicine , Ministry of Education , College of Chemistry and Molecular Sciences , Wuhan University , Wuhan , P. R. China . Email: yuanquan@whu.edu.cn; b Department of Laboratory Medicine & Center for Gene Diagnosis , Zhongnan Hospital , Wuhan University , Wuhan , P. R. China; c Molecular Science and Biomedicine Laboratory , State Key Laboratory of Chemo/Bio-Sensing and Chemometrics , College of Biology and College of Chemistry and Chemical Engineering , Hunan University , Changsha , P. R. China; d Department of Chemistry , Center for Research at the Bio/Nano Interface , Health Cancer Center , UF Genetics Institute , McKnight Brain Institute , University of Florida , Gainesville , USA

## Abstract

Development of a portable device for the detection of multiple mRNAs is a significant need in the early diagnosis of cancer. We have designed a biochip-based mRNA detection device by combining a hydrophilic–hydrophobic micropattern with upconversion luminescence (UCL) probes. The device achieves highly sensitive detection, using the naked eye, of multiple mRNAs among patient samples. The high sensitivity is attributed to enrichment of the target concentration and a fluorescence enhancement effect. In addition, since the photonic crystal (PC) dot biochip is functionalized with dual-wavelength excitation UCL probes, two kinds of mRNAs in the heterogeneous biological samples are detected simultaneously, and the corresponding luminescence signals are captured using an unmodified camera phone. The biochip-based mRNA detection device reported here demonstrates that multiple mRNAs extracted from patient samples can be simultaneously and sensitively detected in a visual way without sophisticated instrumentation. Therefore, this device is promising for real-time detection of multiple biomarkers in patient samples, and it is anticipated that it will provide a powerful tool for convenient early diagnosis of cancer.

## Abstract 2

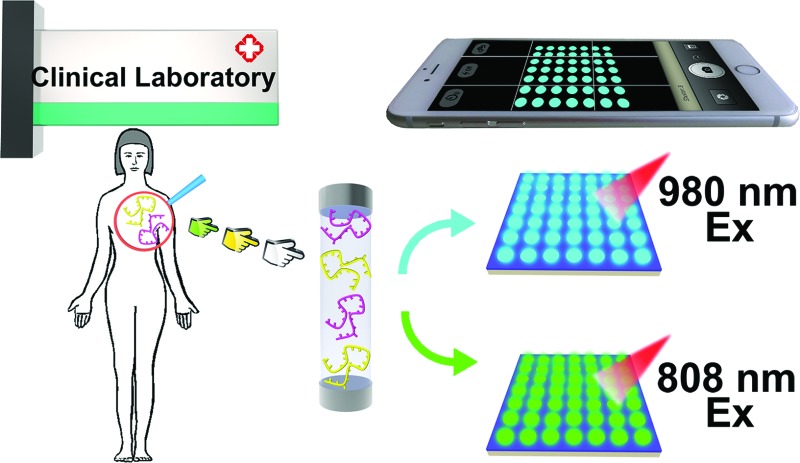
We have designed a biochip-based mRNA detection device by combining a hydrophilic–hydrophobic micropattern with upconversion luminescence (UCL) probes.

## Introduction

The identification of potential cancer biomarkers in patient samples is a key factor in the early diagnosis of cancer.^1–3^ The occurrence of cancer is closely associated with the abnormal expression of genes, and tumor-related mRNA has been commonly used as a specific biomarker to assess the cancer development stage.^4,5^ In recent years, tremendous advances in the field of nucleic acid testing technology offer valuable diagnostic and prognostic approaches for cancer management.^6–12^ Among them, the use of biochip-based devices for mRNA detection is an emerging assay in clinical diagnostic fields.^13,14^ Compared to detection methods using solution systems, biochip-based diagnostics offer the most promising approach for the detection of cancer for point-of-care (POC) applications due to their portability, flexibility, and short sample processing time.^15–17^ According to statistics from the article titled “The Worldwide Market For *In Vitro* Diagnostic (IVD) Tests”, worldwide *in vitro* diagnostics (IVD) market investments are growing every year, indicating that medical diagnostic tools are playing an increasingly important role in human health assessment and disease diagnosis.^18^ The ultimate goal of these endeavors is the development of POC diagnostics with the requisite sensitivity, accuracy, and real-time visualization for patient sample analysis.^13,19^ However, most current biochip-based mRNA detection devices cannot simultaneously fulfill these requirements, and no multiplexed biochip has yet shown direct visual detection of multiple mRNAs in patient samples.^20,21^ These challenges have limited the clinical application of biochip devices in multiple marker analysis and further impede their implementation as effective cancer diagnostic systems. Thus, in order to promote the potential clinical utility of such biochip-based devices, more versatile and robust diagnostic devices that satisfy the clinical requirements are needed for practical patient sample assays.

Recent innovations in optical detection devices with a fluorescence readout have enabled new technological breakthroughs in biomarker analysis.^22–24^ However, because of the low concentration of mRNA in crude patient samples, improving the sensitivity of detection devices is an overriding requirement. This can be accomplished in two ways: one method is to enrich the target substance in the highly dilute solution to obtain a detectable concentration, and the other is to improve the output signal of the detection device. Taking inspiration from enrichment phenomena in nature (*e.g.*, beetles collect fog using a hydrophilic–hydrophobic pattern structure on their backs), the strategy of enriching targets from dilute solutions is a promising means to raise the analyte concentration.^25,26^ Recently, Song and Li have demonstrated well that a hydrophilic–hydrophobic patterned sensor possessed great ability to enrich the target and thus improved the detection sensitivity.^27,28^ On the other hand, with regard to enhancing the output signal of the optical device, fluorescence enhancement can lead to a high signal-to-noise ratio and a lower detection limit. Three-dimensional photonic crystals (PCs) are periodic dielectric materials that can confine, control, and manipulate photons.^29–32^ They have been frequently used to enhance the intensity of some optical species, resulting in a hundred-fold enhancement of the sensitivity.^27^ Overall, through the combined effects of target concentration enrichment and fluorescence enhancement, the sensitivity for mRNA detection in patient samples can be significantly improved.

Cancer is associated with the abnormal expression of multiple tumor-related mRNAs.^33^ A diagnostic device with the ability to detect multiple mRNAs simultaneously is required to avoid false positive results, thus improving the reliability of early cancer diagnosis.^34^ Lanthanide-doped upconversion nanoparticles (UCNPs) that convert near-infrared (NIR) excitation light into shorter wavelength luminescence have recently been widely used as biological probes due to their unique optical properties, such as the absence of autofluorescence, greater light penetration depths and a high resistance to photobleaching.^35–37^ However, while UCNPs can afford tunable multicolored upconversion luminescence (UCL) through control of the lanthanide ion dopants, they typically result in color crosstalk using the same excitation wavelength,^38,39^ which limits the sensitivity and accuracy of multi-analyte detection. Most recently, as an exciting new class of nanophosphors that convert 808 nm NIR light into shorter wavelength luminescence, Nd^3+^ ion-doped UCNPs have attracted great attention for biosensing and imaging.^40–42^ Simultaneous detection of multiple mRNAs can be readily realized using a combination of 808 nm and 980 nm excited UCNPs to avoid color crosstalk between the different labelling signals and allow visual detection to be achieved.

In this work, a biochip-based mRNA detection device with a hydrophilic–hydrophobic micropattern is designed to achieve highly accurate and sensitive detection of multiple mRNAs among patient samples with the naked eye. This portable visual technique provides a powerful tool for convenient cancer diagnosis through the fixing of two kinds of specific UCNP-based mRNA probes on the PC substrates. Our novel mRNA detection device can achieve sensitive, visual detection of multiple mRNAs. The excellent performance of our mRNA detection device could lead to further development into a clinical diagnostic device. This strategy can be extended to design a universal detection device, and it is anticipated that this general method will find a wide-range applications in the areas of health assessment and disease diagnosis.

## Results and discussion

**Fig. 1:**
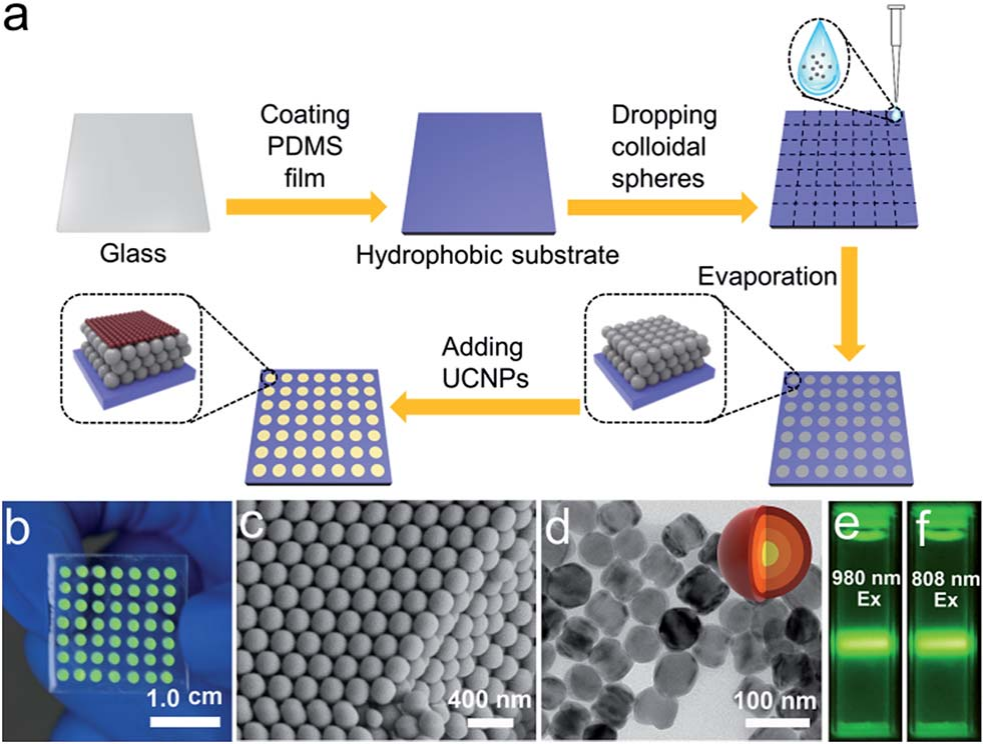
(a) Schematic illustration of fabrication of the PC dot-based substrate. (b) Photograph of the PC-based substrate under exposure to sunlight. (c) SEM image of the PC dots. (d) TEM image of the Er-doped UCNPs. Inset: the structure of the core–shell structured UCNPs. Luminescence photographs of the Er-doped UCNPs using 980 nm (e) and 808 nm (f) illumination with an excitation power density of 1.00 W cm^–2^. The Er-doped UCNPs are dispersed in water.

We sought to generate a sensitive mRNA detection device for bioanalysis of mRNA that is (i) straightforward to fabricate, (ii) sensitive when presented with heterogeneous biological samples, and (iii) convenient for reading of the assay result without any major instrumentation. To satisfy these requirements, a PC dot-based substrate was fabricated by depositing hydrophilic PC dots on a hydrophobic surface. As illustrated in Fig. 1a, aqueous colloidal droplets containing carboxyl-modified polystyrene spheres were dropped onto a specific planned area of the hydrophobic PDMS substrate. Then, concentration led to the formation of macroscopic PC dots during solvent evaporation, and finally UCNPs were added to the PC dots. As shown in Fig. 1b, the aqueous colloidal droplets were assembled into an orderly PC dot array, which appeared green due to the Bragg scattering effect, *via* an evaporation-induced procedure on the hydrophobic substrate. Scanning electron microscopy (SEM) demonstrated that the PC dot array was regularly assembled from the monodispersed polystyrene (PS) spheres (Fig. 1c) with diameters of 215 nm (Fig. S1†). To fabricate efficient optical probes, the core–shell structured β-NaYF_4_:Yb,Er@NaYF_4_:Yb@NaNdF_4_:Yb@NaYF_4_:Yb (denoted as Er-doped UCNPs) with dual NIR excitation wavelengths (808 nm and 980 nm) and the core–shell structured β-NaYF_4_:Yb,Tm@NaYF_4_:Yb (denoted as Tm-doped UCNPs) were employed as light-emitting materials.^43^ The transmission electron microscopy (TEM) images (Fig. 1d and S2†) and the size distribution statistics (Fig. S3†) indicate that the Er-doped UCNPs were constructed from epitaxial layer by layer growth and the as-prepared core–shell UCNPs exhibited a uniform size with diameters of approximately 65 nm. The TEM images (Fig. S4†) of the Tm-doped UCNPs showed that the nanoparticles had well-defined hexagonal shapes, and the mean diameter was found to be approximately 60 nm (Fig. S5†). X-ray powder diffraction (XRD) patterns of the Er-doped UCNPs (Fig. S6†) and Tm-doped UCNPs (Fig. S7†) showed that the diffraction lines could be ascribed to the hexagonal structure of NaYF_4_ with the positions and intensities of the peaks in good agreement with the calculated values for hexagonal-NaYF_4_. The upconversion properties were examined using continuous wave (CW) laser excitation at 980 nm and 808 nm. As shown in Fig. 1e and f, in both cases, the Er-doped UCNPs produced an intense green luminescence when they were doped with Nd^3+^ ions. Remarkably, due to efficient suppression of surface-related deactivation, a large enhancement of the UCL was obtained after simply coating the outermost shell (Fig. S8 and S9†), which could promote efficient upconversion emission for imaging applications. The Tm-doped UCNPs showed a blue emission band at 477 nm using CW laser excitation at 980 nm (Fig. S10†).

**Fig. 2:**
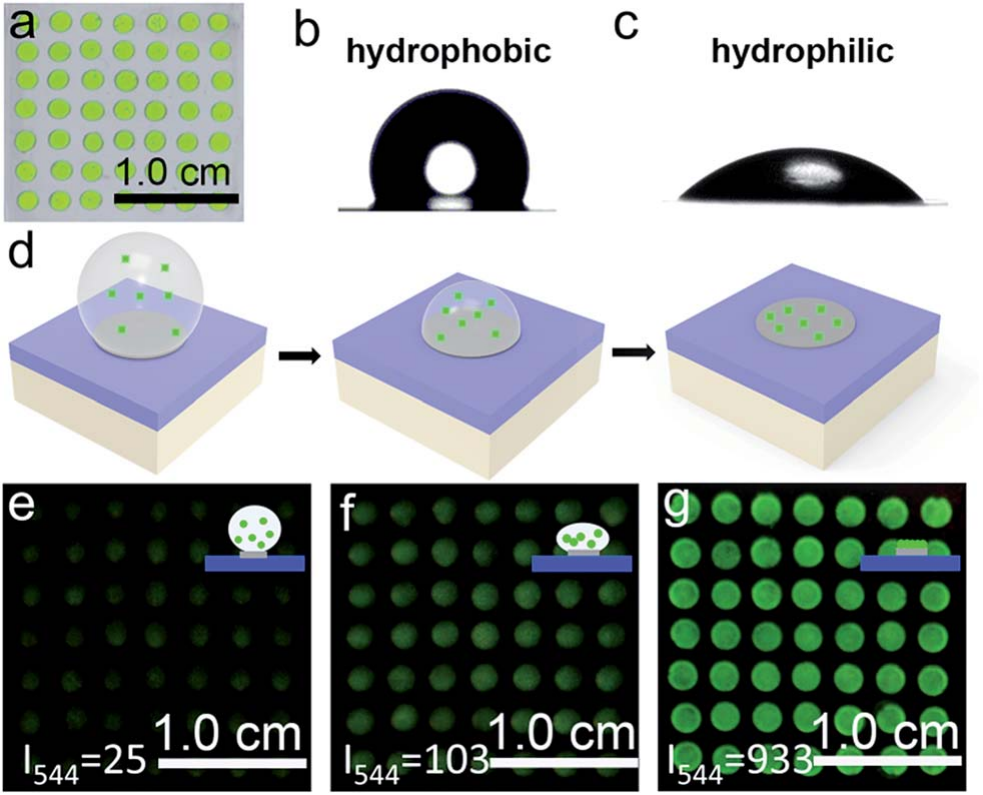
(a) Photograph of the PC dot micropattern with hydrophilic PC dots on the hydrophobic PDMS surface. (b & c) Contact angle characterization of the PDMS-based hydrophobic surface (b) and the PC dot substrate (c). (d) Illustration of the gradual enrichment process of the UCNPs, moving from a highly dilute solution to the PC dot arrays. (e–g) Luminescence images of the PC dot micropattern with the UCNP solution during the condensing–enriching process with 808 nm illumination at an excitation power density of 0.50 W cm^–2^. Inset: sketch of the condensed state.

The PC dot micropattern was fabricated by assembling hydrophilic PC dots on a polydimethylsiloxane (PDMS) hydrophobic substrate. A colloidal solution containing monodispersed PS spheres self-assembled into a regular array on the hydrophobic surface and showed a bright green color when exposed to sunlight (Fig. 2a). The PDMS-coated glass displayed highly hydrophobic behavior with a contact angle of 117.3° ± 2.3° (Fig. 2b), while the contact angle of the PC dot array was 32.3° ± 2.1° and the array resulted in a hydrophilic surface (Fig. 2c). Because of the different wettability characteristics between the PDMS-based substrate and the PC dots, a highly dilute solution of substances could condense onto the hydrophilic PC dots. As illustrated in Fig. 2d, a solution of UCNPs was first dropped onto the PC dots. With evaporation of the water, the solution dewet from the hydrophobic substrate and the UCNPs were condensed on the PC dots. Herein, the images of the luminescence intensities were clearly obtained using an unmodified camera phone. At the beginning, the luminescence image of the UCNPs deposited on the PC dots was dark due to the highly dilute solution of UCNPs (Fig. 2e), and then the UCL intensity increased after evaporation of part of the water (Fig. 2f). When the water had dried, the UCNPs deposited on the PC dots became much brighter due to complete enrichment of the UCNPs (Fig. 2g). The corresponding luminescence spectra of the PC dots also showed that the visible emission light of the PC dots became brighter and brighter during the enrichment process (Fig. S11†). Compared to the UCL intensity of the UCNPs on a PC dot, the luminescence intensity from a droplet containing the same number of UCNPs on a pure hydrophilic PC film after evaporation declined by half (Fig. S12†), which further confirmed the great enrichment ability of the PC dot-based substrate with a hydrophilic–hydrophobic pattern.

**Fig. 3:**
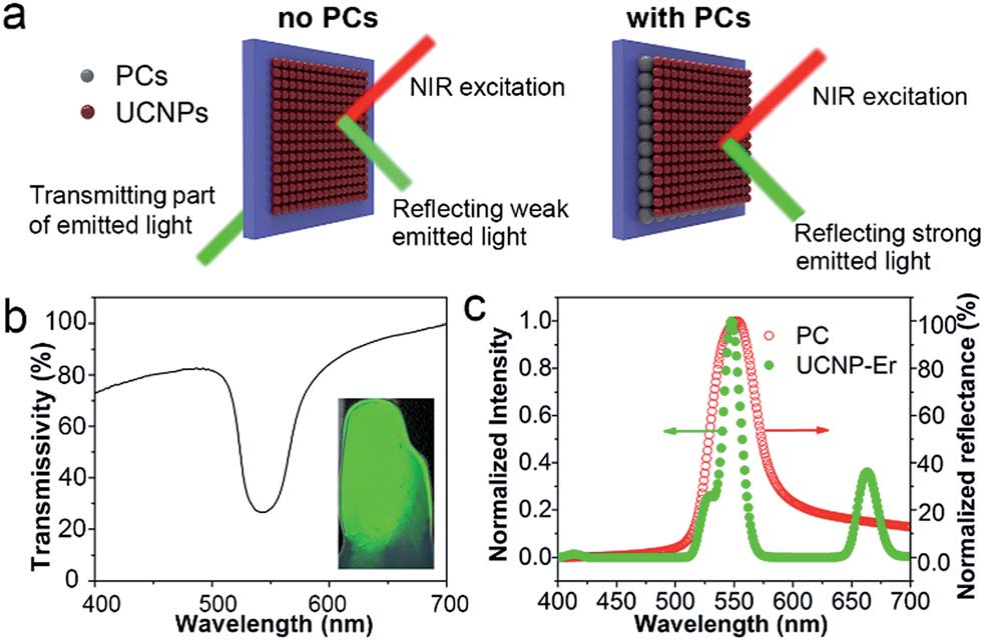
(a) Optical pathway diagrams of the upconversion emitted light when Er-doped UCNPs are deposited on the substrate without (left) and with (right) PCs under NIR excitation. (b) Transmittance spectrum of the PC film. Inset: photograph of the PC film. (c) Reflection spectrum of the PC film, together with a normalized luminescence spectrum of the Er-doped UCNPs dispersed in water under 808 nm excitation.

Due to their photonic band-gap properties, three-dimensional PC materials can be used to modulate the emission wavelength and intensity of optical species.^44^ In particular, enhanced luminescence can be obtained when UCNPs are combined with PCs because of enhanced reflection of the emitted light.^45^ As illustrated in Fig. 3a, without PCs, only part of the emitted light of the UCNPs can be reflected and most is transmitted through the glass substrate, thus the luminescence of the UCNPs is not enhanced. However, when the glass substrate is coated with PC dots, almost all of the emitted light is reflected. Since the PCs can efficiently prevent the emitted light from being transmitted through the substrate, the luminescence signal of the UCNPs is readily enhanced. As shown in the transmittance spectrum in Fig. 3b, the stopband of the PC film was located at 552 nm. The inset image in Fig. 3b shows the green color present under exposure to sunlight due to the Bragg scattering effect. Also, as shown in Fig. 3c, the reflection spectrum of the PC film showed a stopband centered at 552 nm. The Er-doped UCNPs showed a green emission band at 544 nm under NIR excitation, which overlaps well with the stopband of the PC. This result indicates that the photonic gap of the PCs can result in efficient reflection of the green emission band at 544 nm and prevent transmission of the emitted light, leading to an enhancement of the UCL.

**Fig. 4:**
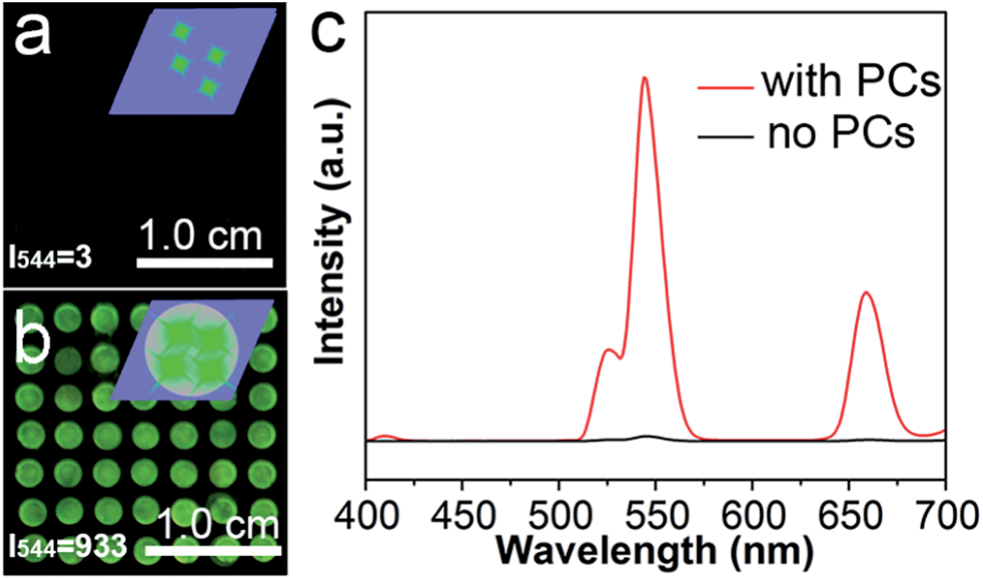
Luminescence images of the Er-doped UCNPs deposited on the substrate without (a) and with (b) PC dots using an excitation power density of 0.50 W cm^–2^ and 808 nm light. Inset: illustration of the UCNPs deposited on the substrate. (c) UCL spectra of the Er-doped UCNPs on the substrate with and without PCs. *I*_544_ is the UCL intensity of the emission band at 544 nm.

The enhancement ability of the PC dots when using UCNPs was investigated by comparing the luminescence intensities of the UCNPs on different substrates: a substrate without PC dots and a substrate with PC dots. It can be noted that the luminescence image in Fig. 4a is dark, as there were no PC dots on the substrate. However, when the UCNPs were loaded on the substrate with PC dots, the luminescence image became bright (Fig. 4b). The enhanced luminescence intensity of the UCNPs was attributed to an optical enhancement effect of the PCs. These luminescence photographs clearly suggest that the PC dot substrate displays a powerful ability to enhance the luminescence of UCNPs. The corresponding luminescence spectra (Fig. 4c) also show that the luminescence intensity of the UCNPs on the PC dot substrate was much stronger than that for the substrate without PCs. These results prove that the PC dot substrate is an efficient tool for enhancing the luminescence intensity of UCNPs.

**Fig. 5:**
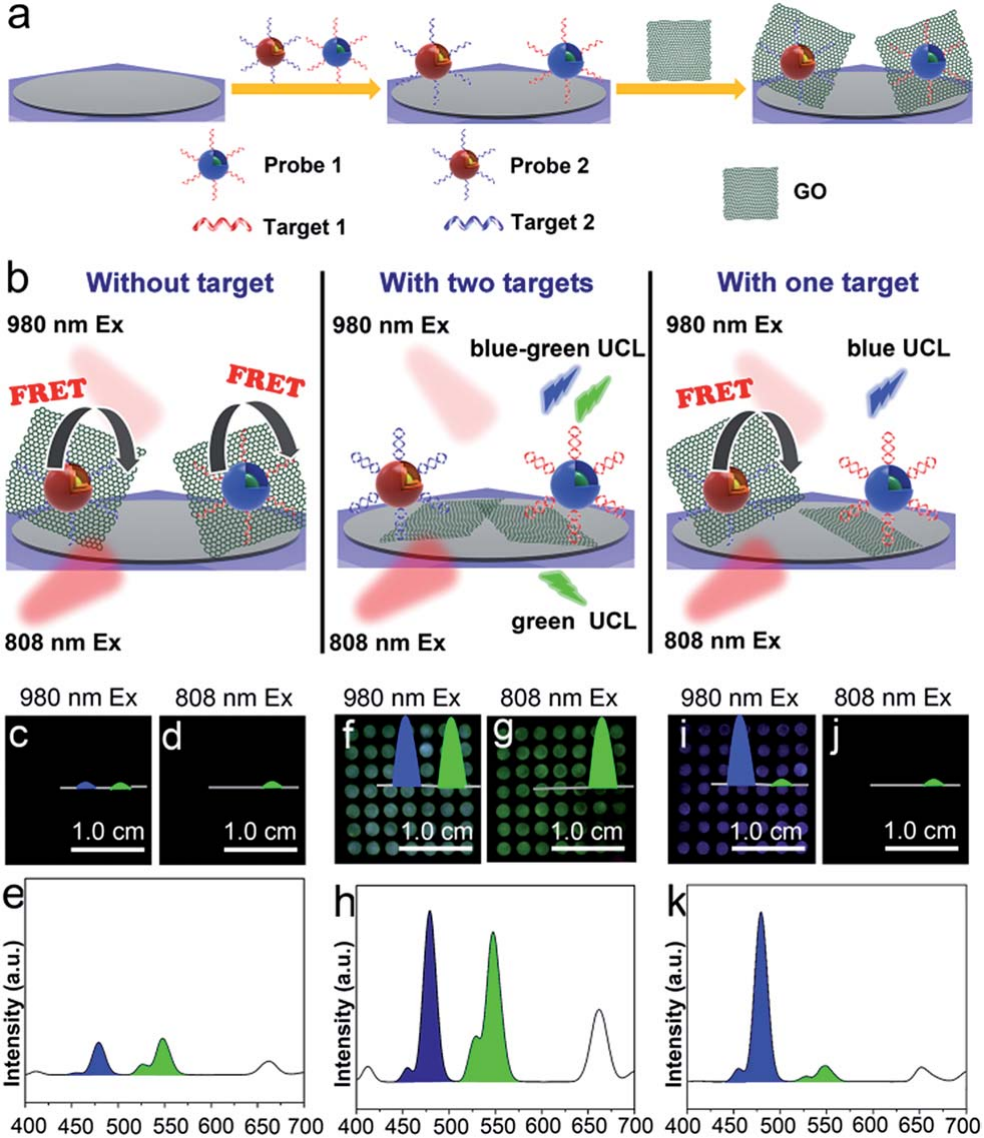
(a) Schematic illustration of the construction of a flexible detection device. (b) Working principle for the simultaneous detection of multiple mRNAs. (c & d) Luminescence images of the biochip-based detection device without mRNA using 980 nm (c) and 808 nm (d) excitation. Insets: diagrams of the UCL bands at 477 and 544 nm. (e) UCL spectrum of the detection device without mRNA. (f & g) Luminescence images of the detection device with the addition of TK1 and C-myc mRNA using 980 nm (f) and 808 nm (g) excitation. Insets: diagrams of the UCL bands at 477 and 544 nm. (h) UCL spectrum of the detection device with the addition of TK1 and C-myc mRNA. (i & j) Luminescence images of the detection device when only the C-myc mRNA was present using 980 nm (i) and 808 nm (j) excitation. Insets: diagrams of the UCL bands at 477 and 544 nm. (k) UCL spectrum of the detection device with the addition of only C-myc mRNA. The excitation power density of the 980 nm and 808 nm CW laser was 0.50 W cm^–2^.

According to previous studies, mRNAs are proposed to be important markers for tumor growth and they are usually chosen for assessing the stage of cancer development.^46^ However, it has been reported that a specific kind of tumor may correlate with multiple mRNA markers, and that some tumor-related mRNAs are also expressed in normal tissues. Thus, if more than one kind of tumor-related mRNA exists, the diverse expression levels of these mRNAs should all be detected to improve the reliability of the diagnosis. Fig. 5a illustrates the construction of a flexible device for the simultaneous detection of multiple tumor biomarkers. Specifically, different kinds of UCNP-based probes (two probes, named probe 1 and probe 2, were used as an example) are constructed by labelling the recognition sequences of different target mRNAs (target 1 and target 2 were used as examples) with Er-doped UCNPs (*λ*_exc_ = 808 nm, *λ*_em_ = 544 nm) and Tm-doped UCNPs (*λ*_exc_ = 980 nm, *λ*_em_ = 477 nm), respectively. The detection device is composed of the PC dot substrate, UCNP-based probes and graphene oxide (GO). The UCNP-based probes are immobilized on the PC dot substrate and then they bind to GO *via* π–π stacking between the nucleobases of the recognition sequences and the sp^2^ bonded carbon atoms of GO. The detection of two kinds of mRNA using the above device is illustrated in Fig. 5b and S14a.† When neither target is present, the luminescence is very weak due to quenching of the UCL by GO. In the presence of the two targets, the two kinds of probes hybridize with their corresponding targets, leading to recovery of the luminescence signal due to detachment of the GO from the two kinds of probes. In the presence of one of the targets, only the corresponding probe hybridizes with the target and its luminescence signal is recovered. Previous studies have reported that TK1 mRNA and C-myc mRNA are highly correlated with the development of breast cancer,^43^ thus these two kinds of mRNA were chosen to test the biosensing capabilities of the device. In the presence of an increasing amount of GO, the UCL at 477 nm and 544 nm of the probes decreased gradually when using 980 nm excitation, indicating that the UCL can be effectively quenched by GO (Fig. S13†). The luminescence images of the detection device without the mRNA added were dark, using both 980 nm (Fig. 5c) and 808 nm (Fig. 5d) excitation, due to the weak visual emission bands at 477 nm and 544 nm (Fig. 5e). With the simultaneous addition of TK1 mRNA and C-myc mRNA, the device exhibited a blue-green luminescence consisting of green and blue bands, using 980 nm laser illumination (Fig. 5g). The UCL spectrum with excitation at 980 nm showed that the luminescence intensities at both 477 nm and 544 nm were enhanced when the two kinds of mRNA were present simultaneously (Fig. 5h). When only one kind of mRNA target (*e.g.*, C-myc mRNA) was present, the device emitted blue luminescence, using excitation at 980 nm (Fig. 5i). The test zone became dark when the wavelength of the CW laser was changed to 808 nm, since the UCL of the Tm-doped UCNPs cannot be obtained using excitation at 808 nm (Fig. 5j). The corresponding luminescence spectrum also demonstrates that only the blue luminescence triggered by C-myc mRNA was observed (Fig. 5k). Similarly, in the presence of only TK1 mRNA, the detection device exhibited green luminescence under both 980 nm (Fig. S14b†) and 808 nm (Fig. S14c†) excitation because the Er-doped UCNPs can be excited at both 980 nm and 808 nm. As a result, this mRNA detection device exhibited good selectivity during the simultaneous detection of two kinds of mRNA. In addition, the UCL intensity of the PC dots gradually weakened when the concentrations of TK1 mRNA and C-myc mRNA were decreased from 0.1 nM to 0.01 nM and the detection limit was determined to be 0.01 nM (Fig. S15 and S16†), demonstrating that the detection device also exhibits high sensitivity for the detection of mRNA. The luminescence from the mRNA detection device was visible to the naked eye and could be captured using an unmodified camera phone. The above results therefore indicate that the mRNA detection device is capable of simultaneous and sensitive detection of multiple mRNAs using the naked eye.

**Fig. 6:**
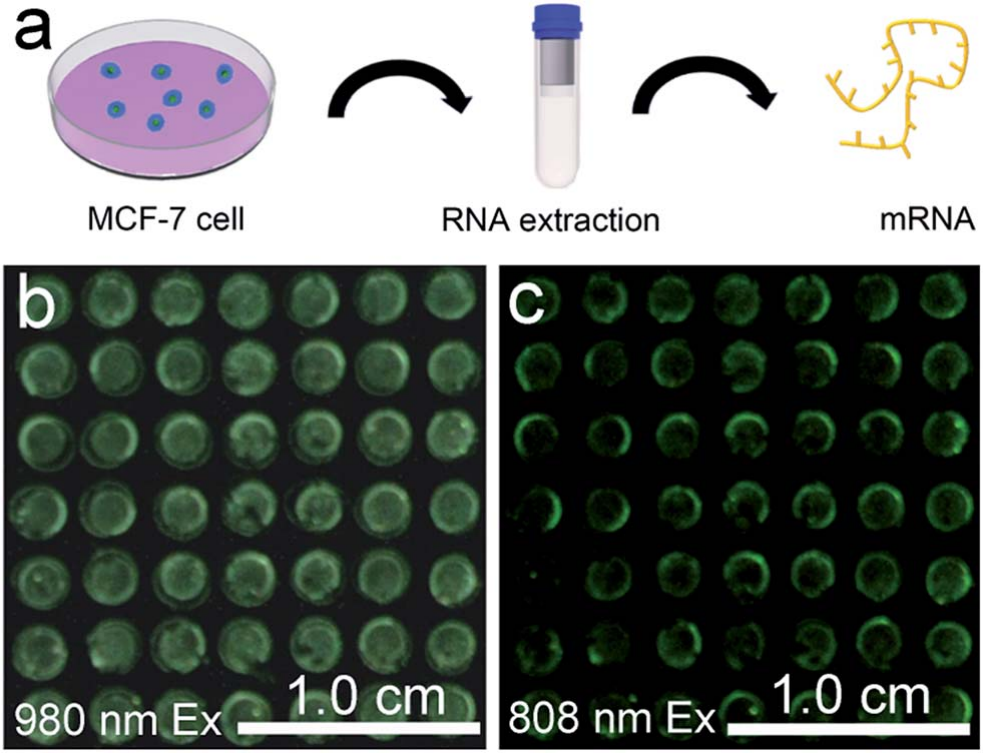
(a) Schematic illustration of the mRNA extraction from MCF-7 cells. Luminescence images of the mRNA detection device with the addition of targets extracted from the MCF-7 cells, with 980 nm (b) and 808 nm (c) illumination at an excitation power density of 0.50 W cm^–2^.

The favourable performance of the mRNA detection device indicated promising prospects for assay of the mRNAs present in heterogeneous biological samples. MCF-7 is a human breast adenocarcinoma cell line, where TK1 and C-myc mRNA are overexpressed. mRNAs isolated from the MCF-7 cells were detected to preliminarily determine whether the device is robust enough for analysis of biological samples (Fig. 6a). In the presence of mRNAs extracted from 10^5^ MCF-7 cells, the device exhibited a blue-green luminescence consisting of green and blue bands using 980 nm laser illumination (Fig. 6b). In addition, the device exhibited green luminescence under 808 nm excitation (Fig. 6c). The luminescence spectra showed that in comparison to the UCL intensity of the device without mRNA extracts, stronger visual emission bands at 477 nm and 544 nm were obtained under 980 nm excitation when there existed mRNAs extracted from MCF-7 cells (Fig. S17†). The mRNA detection device is therefore highly sensitive in the detection of crude biological samples.

**Fig. 7:**
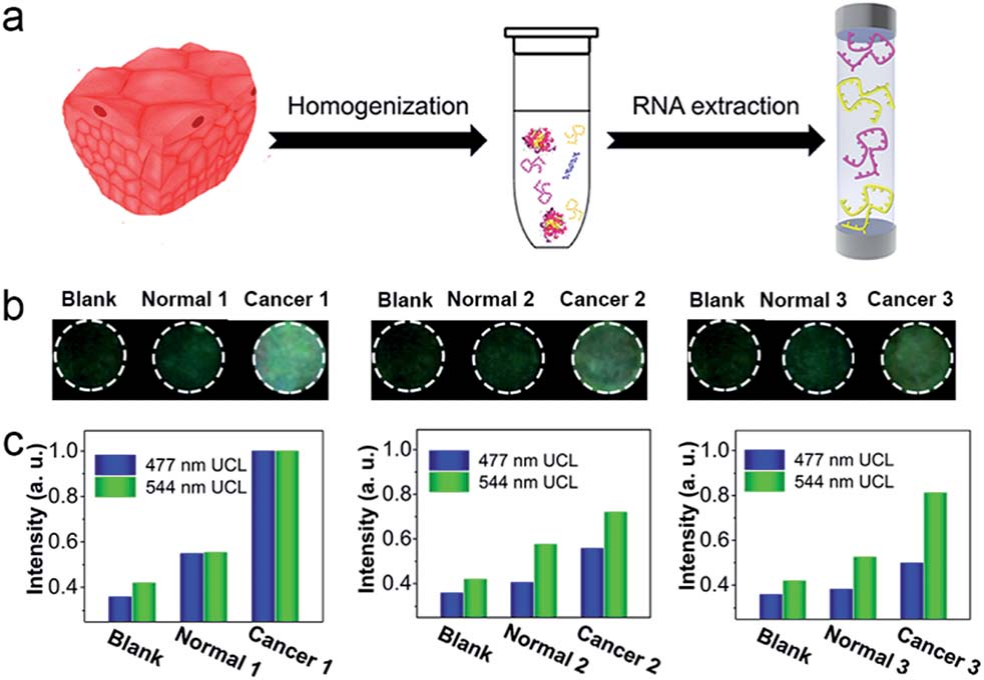
(a) Schematic illustration of the mRNA extraction from patient samples. (b) Luminescence images of the mRNA detection device using 980 nm illumination at an excitation power density of 0.50 W cm^–2^. Blank: without RNA extracts. Normal: with RNA extracts from normal tissue. Cancer: with RNA extracts from cancer tissue. (c) The luminescence intensities of the mRNA detection device with the addition of patient samples containing normal tissue and cancer tissue.

To investigate the applicability of this device for clinical diagnosis, mRNAs extracted from breast cancer patient samples were assayed. Since the expression levels of certain mRNAs are accurate predictors of the patient's overall prognosis, mRNAs from breast cancer tissues were extracted to investigate the expression levels of the corresponding mRNAs, as shown in Fig. 7a. Also, mRNAs from a corresponding non-cancerous normal tissue sample from the same patient were extracted. As show in Fig. 7b, three breast cancer patient samples containing cancerous tissue and the corresponding non-cancerous normal tissues were detected. The detection device without RNA extracts was employed as a blank, and it showed dark images using 980 nm laser illumination. With the mRNAs extracted from the normal tissues, the luminescence images of the detection device were slightly brighter than for the blank group, indicating that normal tissue expresses low levels of TK1 and C-myc mRNA. Furthermore, in the presence of the mRNAs extracted from the breast cancer tissues, the detection areas of the devices all exhibited a bright blue-green luminescence consisting of green and blue luminescence bands, using 980 nm laser illumination. Clearly, with the addition of mRNAs extracted from breast cancer tissue, the detection devices exhibited a significantly much stronger luminescence than for the group with mRNAs extracted from normal tissue, and these effects were detected with the naked eye. It can be noted that similar results were obtained when the three patient samples were assayed simultaneously, demonstrating that TK1 mRNAs and C-myc mRNAs are all overexpressed in breast tumor tissue, but expressed at relatively low levels in normal tissue. Corresponding luminescence spectra of the detection devices were obtained, as shown in Fig. 7c, and the UCL intensities from the breast cancer tissues were much higher than those of the normal tissue samples and the blank group. These results demonstrate that our device holds great promise for cancer diagnostics.

## Conclusions

In conclusion, the new mRNA detection device described herein was able to detect tumor-related mRNAs directly in clinically relevant samples, using the luminescence signals of UCNPs for the first time. The biochip-based mRNA detection device with a hydrophilic–hydrophobic pattern showed both a target enrichment ability and luminescence enhancement simultaneously, thus leading to sensitive detection of mRNAs extracted from patient samples using the naked eye. More importantly, since distinct excitation–emission peaks were obtained through irradiation with two CW lasers at wavelengths of 980 nm and 808 nm, the detection device exhibited good accuracy for the simultaneous detection of two kinds of mRNA, and false positive results were successfully avoided. The results from the luminescence images captured using an unmodified camera phone proved that such a detection device was capable of assaying patient samples without sophisticated instrumentation. This multifunctional device shows great potential for the early diagnosis of cancer and is anticipated to find extensive applications in clinical diagnosis and life science.

## References

[cit1] Wu L., Qu X. G. (2015). Chem. Soc. Rev..

[cit2] Karachaliou N., Mayo-de-las-Casas C., Molina-Vila M. A., Rosell R. (2015). Ann. Transl. Med..

[cit3] Li N., Chang C. Y., Pan W., Tang B. (2012). Angew. Chem., Int. Ed..

[cit4] Hanahan D., Weinberg R. A. (2000). Cell.

[cit5] Peng X. H., Cao Z. H., Xia J. T., Carlson G. W., Lewis M. M., Wood W. C., Yang L. (2005). Cancer Res..

[cit6] Seferos D. S., Giljohann D. A., Hill H. D., Prigodich A. E., Mirkin C. A. (2007). J. Am. Chem. Soc..

[cit7] Raj A., Bogaard P., A Rifkin S., Oudenaarden A., Tyagi S. (2008). Nat. Methods.

[cit8] Lin M. H., Wang J. J., Zhou G. B., Wang J. B., Wu A., Lu J. X., Gao J. M., Chen X. Q., Shi J. Y., Zuo X. L., Fan C. H. (2015). Angew. Chem., Int. Ed..

[cit9] Qiu L. P., Wu C. C., You M. X., Han D., Chen T., Zhu G. Z., Jiang J. H., Yu R. Q., Tan W. H. (2013). J. Am. Chem. Soc..

[cit10] Buxbaum A. R., Wu B., Singer R. H. (2014). Science.

[cit11] Duan R. X., Zuo X. L., Wang S. T., Quan X. Y., Chen D. L., Chen Z. F., Jiang L., Fan C. H., Xia F. (2013). J. Am. Chem. Soc..

[cit12] Zhang K., Zhu X., Jia F., Auyeung E., Mirkin C. A. (2013). J. Am. Chem. Soc..

[cit13] Choi N. W., Kim J., Chapin S. C., Duong T., Donohue E., Pandey P., Broom W., Hill W. A., Doyle P. S. (2012). Anal. Chem..

[cit14] Wu Y., Kwak K. J., Agarwal K., Marras A., Wang C., Mao Y., Huang X., Ma J., Yu B., Lee R., Vachani A., Marcucci G., Byrd J. C., Muthusamy N., Otterson G., Huang K., Castro C. E., Paulaitis M., Nana-Sinkam S. P., Lee L. J. (2013). Anal. Chem..

[cit15] Gervais L., de Rooij N., Delamarche E. (2011). Adv. Mater..

[cit16] Vashist S. K., Lam E., Hrapovic S., Male K. B., Luong J. H. T. (2014). Chem. Rev..

[cit17] Bourquin Y., Syed A., Reboud J., Ranford-Cartwright L. C., Barrett M. P., Cooper J. M. (2014). Angew. Chem., Int. Ed..

[cit18] The Worldwide Market For In Vitro Diagnostic (IVD) Tests, 6th Edition [with 2009 Economy Preface], Kalorama Information, 2008.

[cit19] Sassolas A., Leca-Bouvier B. D., Blum L. J. (2008). Chem. Rev..

[cit20] Fang Z., Soleymani L., Pampalakis G., Yoshimoto M., Squire J. A., Sargent E. H., Kelley S. O. (2009). ACS Nano.

[cit21] Liu Y. H., Yao H. X., Zhu J. (2013). J. Am. Chem. Soc..

[cit22] McDonagh C., Burke C. S., MacCraith B. D. (2008). Chem. Rev..

[cit23] Manzano J. R., Karymov M. A., Begolo S., Selck D. A., Zhukov D. V., Jue E., Ismagilov R. F. (2016). ACS Nano.

[cit24] Chen W. W., Li Q. Z., Zheng W. S., Hu F., Zhang G. X., Wang Z., Zhang D. Q., Jiang X. Y. (2014). Angew. Chem., Int. Ed..

[cit25] Parker A. R., Lawrence C. R. (2001). Nature.

[cit26] Darmanin T., Guittard F. (2014). J. Mater. Chem. A.

[cit27] Hou J., Zhang H., Yang Q., Li M., Song Y., Jiang L. (2014). Angew. Chem., Int. Ed..

[cit28] Hou J., Zhang H. C., Yang Q., Li M. Z., Jiang L., Song Y. L. (2015). Small.

[cit29] Yablonovitch E. (1987). Phys. Rev. Lett..

[cit30] Scherer A., Painter O., Vuckovic J., Loncar M., Yoshie T. (2002). IEEE Trans. Nanotechnol..

[cit31] Shen W. Z., Li M. Z., Xu L. A., Wang S. T., Jiang L., Song Y. L., Zhu D. B. (2011). Biosens. Bioelectron..

[cit32] Li M. Z., He F., Liao Q., Liu J., Xu L., Jiang L., Song Y. L., Wang S., Zhu D. B. (2008). Angew. Chem., Int. Ed..

[cit33] Sidransky D. (1997). Science.

[cit34] Zhou L., Wang R., Yao C., Li X., Wang C., Zhang X., Xu C., Zeng A., Zhao D., Zhang F. (2015). Nat. Commun..

[cit35] Haase M., Schafer H. (2011). Angew. Chem., Int. Ed..

[cit36] Liu J., Liu Y., Liu Q., Li C., Sun L., Li Y. F. (2011). J. Am. Chem. Soc..

[cit37] Chen G., Qiu H., Prasad P. N., Chen X. Y. (2014). Chem. Rev..

[cit38] Tian G., Gu Z., Zhou L., Yin W., Liu X., Yan L., Jin S., Ren W., Xing G., Li S., Zhao Y. (2012). Adv. Mater..

[cit39] Wu S., Duan N., Shi Z., Fang C., Wang Z. (2014). Anal. Chem..

[cit40] Xie X., Gao N., Deng R., Sun Q., Xu Q. H., Liu X. G. (2013). J. Am. Chem. Soc..

[cit41] Wang Y. F., Liu G. Y., Sun L. D., Xiao J. W., Zhou J. C., Yan C. H. (2013). ACS Nano.

[cit42] Li X., Guo Z., Zhao T., Lu Y., Zhou L., Zhao D., Zhang F. (2016). Angew. Chem., Int. Ed..

[cit43] Liu B., Chen Y. Y., Li C. X., He F., Hou Z. Y., Huang S. S., Zhu H. M., Chen X. Y., Lin J. (2015). Adv. Funct. Mater..

[cit44] Yin Z., Zhu Y. S., Xu W., Wang J., Xu S., Dong B., Xu L., Zhang S., Song H. W. (2013). Chem. Commun..

[cit45] Liao J. L., Yang Z. W., Wu H. J., Yan D., Qiu J. B., Song Z. G., Yang Y., Zhou D. C., Yin Z. Y. (2013). J. Mater. Chem. C.

[cit46] Robertson J. F. R., ONeill K. L., Thomas M. W., McKenna P. G., Blamey R. W. (1990). Br. J. Cancer.

