# Supplementary material for: Naked eye detection of multiple tumor-related mRNAs from patients with photonic-crystal micropattern supported dual-modal upconversion bioprobes
Source: Chem Sci. 2016 Aug 19;8(1):466–72. doi: 10.1039/c6sc03401b (PMC5458711; doi:10.1039/c6sc03401b)
Supplement: Supplementary file 1 [file SC-008-C6SC03401B-s001.pdf]

Electronic Supplementary Material (ESI) for Chemical Science.
This journal is © The Royal Society of Chemistry 2016

Supporting Information

Naked Eye Detection of Multiple Tumor-Related mRNAs from Patients with Photonic-Crystal Micropattern Supported Dual-Modal Upconversion Bioprobes

Xiaoxia Hu[a], Yingqian Wang[a], Haoyang Liu[a], Jie Wang[a], Yaning Tan[a], Fubing Wang[b], Quan Yuan*[,a], and Weihong Tan[c,d]

[a]Key Laboratory of Analytical Chemistry for Biology and Medicine (Ministry of Education), College of Chemistry and Molecular Sciences, Wuhan University, Wuhan, P. R. China
[b]Department of Laboratory Medicine & Center for Gene Diagnosis, Zhongnan Hospital, Wuhan University, Wuhan, P. R. China
[c]Molecular Science and Biomedicine Laboratory, State Key Laboratory of Chemo/Bio-Sensing and Chemometrics College of Biology and College of Chemistry and Chemical Engineering, Hunan University, Changsha, P. R. China
[d]Department of Chemistry, Center for Research at the Bio/Nano Interface, Health Cancer Center, UF Genetics Institute, McKnight Brain Institute, University of Florida, Gainesville, USA
*Corresponding author: yuanquan@whu.edu.cn

Experimental Details

Materials and reagents. Yttrium oxide (Y_2O_3, 99.99%), ytterbium oxide (Yb_2O_3, 99.99%), thulium oxide (Tm_2O_3, 99.99%), erbium oxide (Er_2O_3, 99.99%), sodium trifluoroacetate (CF_3COONa, 98%), Poly(ethylenimine) (PEI, branched polymer, average MW 25 kDa), diethylene glycol (DEG), Oleic acid (OA), 1-octadecene (ODE) were purchased from Aladdin. Ethanol (AR), cyclohexane (AR), chloroform (AR), toluene (AR) were received from Sinopharm Chemical Reagent Co. (China). PDMS (Sylgard 184 silicone elastomer kit, Dow), curing agent, monodispersed latex spheres polystyrene (PS), m/v = 10%) were obtained from Dow Corning. Sulfosuccinimidyl-4-(N-maleimidomethyl)cyclohexane-1-carboxylate (Sulfo-SMCC), HEPES buffer solution (4-(2-Hydroxyethyl)-1-piperazineethanesulfonic acid (HEPES)), EDC (1-ethyl-3-[3-dimethyllamino-propyl]carbodiimide hydrochloride), NHS (N-hydroxysuccinimide) were purchased from Sigma Aldrich.

Sample characterization. The morphology of the PEI-coated UCNPs was characterized by a JEM-2100 transmission electron microscope (JEOL) with an accelerating voltage of 200 kV. Zeta potential measurements were performed using a Malvern Zetasizer Nano ZS system. The phase of nanocrystals was carried out using a D8 Advance X-ray diffractometer (Bruker) with a Cu–Kα radiation (λ = 1.5406 Å). UV-vis transmission spectra were obtained on a UV–2550 scanning spectrophotometer (Shimadzu). Luminescence spectra were recorded on a Hitachi F–4600 fluorescence spectrometer equipped with a 980 nm CW laser and an 808 nm laser (Kai Site Electronic Technology Co., Ltd.). Contact angles were captured by OCA20 instrument (Dataphysics, Germany) at 25 °C. Deionized water (MilliQ, 18.5 MΩ cm) was employed as the source for the CA measurement. The full image of the PC microchip was captured by digital camera 60D (Canon Co., Ltd). The luminescence images were captured by an unmodified camera phone.

Synthesis of $RE(CF_3COO)_3$. In a typical preparation, a kind of lanthanide oxide was added into the appropriate amount of water to form a slurry, then slighted excessive trifluoroacetic acid was added into the forementioned slurry under vigorously stirring. The whole solution was kept refluxing under 140 °C until a transparent solution was formed. The resulting solution was transferred into an evaporating basin after a filtered step. With the water evaporated, $RE(CF_3COO)_3$ powders were obtained.

Preparation of the core-shell upconversion nanoparticles (UCNPs). In the present work, two kinds of core-shell UCNPs ($NaYF_4$:Yb,Er@$NaYF_4$:Yb@$NaNdF_4$:Yb@$NaYF_4$:Yb and $NaYF_4$:Yb,Tm@$NaYF_4$:Yb) were chosen as the efficient probes to detect mRNAs. Hexagonal-phase OA-coated core nanoparticles $NaYF_4$:18%Yb,2%Er (or $NaYF_4$:19%Yb,1%Tm) were first obtained using a previously reported protocol.[1] In brief, a specified proportion of $RE(CF_3COO)_3$ (1 mmol), CF_3COONa (1 mmol), and a mixture of OA/ODE (40 mmol, n/n = 1:1) were added into in a three-necked flask, and then were heated to 120 °C for 30 min with magnetic stirring under vacuum. Next, the resulting solution was

heated to 340 °C and was maintained it at 340 °C for 30 min with stirring under an Ar atmosphere. After cooling to the room temperature, the nanoparticles can be obtained through adding excessive amount of ethanol, and then washed three time with ethanol and cyclohexane (v/v = 1:1). Finally, these nanoparticles were dried in the air. For the epitaxial growth of the shell layer, the as-obtained core nanoparticles were dispersed in a mixture of OA/ODE (20 mL, v/v = 1:1) in a 100 mL three-necked flask. Then, the slurry was heated to 120 °C under vacuum with magnetic stirring until the total removal of the residual water and oxygen. Next, the solution was heated at 310 °C under an Ar atmosphere. Meanwhile, a mixture of OA/ODE (4 mL, v/v = 1:1) containing 0.5 mmol of $RE(CF_3COO)_3$ (RE = 90%Y + 10%Yb or 90%Nd + 10%Yb) and 0.5 mmol of CF_3COONa was injected into the hot solution dropwise, then the resulting solution was maintained at 310 °C for 1 h. The step of hot injection was repeated to maintain the layer-by-layer growth of the multi-layers nanoparticles under the corresponding change of the component. Finally, excess absolute ethanol was added into the mixture at the room temperature. The core-shell nanoparticles were obtained after centrifugation.

Synthesis of PEI-functionalized UCNPs. The as-obtained UCNPs should undergo a ligand exchange to be water-soluble from the oleic-acid stabilized UCNPs to the PEI-functionalized UCNPs.[2] In details, 300 mg of polyethylenimine (PEI) and 30 mL of DEG were added into a 100 mL three-necked flask. The mixture was then heated to 110 °C to form a pellucid solution under vacuum. Then a mixture of chloroform/toluene (5 mL, v/v = 2:3) containing 100 mg of as-prepared UCNPs was added drop by drop and then was maintained at 110 °C for 1 h under an Ar atmosphere. Then the resulting solution was heated to 240 °C for 1.5 h. After cooling down to room temperature, the precipitate can be obtained by adding ethanol, followed by centrifugation. Finally, the PEI-UCNPs were dispersed in ultrapure water after washing three times with ethanol and water (v/v = 1:1).

Fabrication of the PC dots-based substrate. The PC dots-based substrate with a hydrophilic−hydrophobic micropattern was successfully fabricated through a solvent evaporation method. In details, PDMS and the curing agent (m/m = 10:1) were first blended, followed by vigorously stirring for 30 min and then was treated under vacuum until there was no bubble any more. Then the mixture of PDMS and curing agent was spin-coated on the clean cover glass, and then the PDMS coated glass was cured in an oven at 60 °C overnight to form a transparent and hydrophobic PDMS substrate. Next, 2 μL of monodispersed latex spheres (polystyrene, PS) suspension was dropped vertically on the PDMS substrate, and then was heated on the heating plate at 40 °C and maintained at this temperature until the PCs droplet was dried. Finally, these prepared substrates were placed in a dry condition for further use.

Measurement of upconversion luminescence (UCL) on the PC dots-based substrate during the enrichment process. The solution of UCNPs (1.0 mg mL^{-1}, 2.0 μL) was first dropped on the PC dot. Then, the PC dots-based substrate with UCNPs solution was irradiated with an 808 nm CW laser at excitation power density of 0.50 W/cm^2 and the luminescence image was captured by an unmodified camera phone. Five minutes later, part of the water evaporated and the luminescence image of the PC dots-based substrate with UCNPs was further captured. After nine minutes, the water dried and UCNPs were condensed on the PC dots. The PC dots-based substrate with UCNPs was irradiated with an 808 nm CW laser at excitation power density of 0.50 W/cm^2 and the luminescence image was captured.

Measurement of UCL on the hydrophilic PC film. The solution of UCNPs (1.0 mg mL^{-1}, 2.0 μL) was dropped on a pure hydrophilic PC film. After three minutes, the water dried and the PC film with UCNPs was irradiated with an 808 nm CW laser at excitation power density of 0.50 W/cm^2, and then the luminescence image was captured by an unmodified camera phone.

Measurement of upconversion luminescence (UCL) on different substrates. Droplets of UCNPs solution (1.0 mg mL^{-1}, 2.0 μL) were firstly dropped on different substates: substrate without PC dots and substrate with PC dots. The water on the substrate without PC dots was dried after ten minutes, while the water on the substrate with PC dots was dried after nine minutes. Then, the luminescence images of the UCNPs-doped substrates without and with PC dots at excitation power density of 0.50 W/cm^2 were captured by an unmodified camera phone.

Preparation of mRNA probes. To prepare mRNA probes, amino-modified Er-UCNPs were functionalized with the recognition sequences of TK1 mRNA (TK1 recognition segment: 5’-NH_2-$(CH_2)_6(A)_9$GCGAGTGTCTTTGGCATACTT$(A)_9$-$(CH_2)_6$-SH-3’), and amino-modified Tm-UCNPs were linked with recognition sequences of C-myc mRNA (C-myc recognition segment: 5’-NH_2-$(CH_2)_6(A)_9$ TTGGTGAAGCTAACGTTGAGG$(A)_9$-$(CH_2)_6$-SH-3’). Sulfo-SMCC as a bifunctional cross-linker was used to conjugate PEI-UCNPs with SH-DNA following the previous protocol.[3] Typically, 0.2 mg of Sulfo-SMCC was added to a homogenous mixture containing 5 mL of PB buffer solution (0.01 M, pH = 7.4) and 2 mg of PEI-UCNPs. After reacting for 1 h, the maleimide-activated UCNPs were recovered by centrifugation and the excess Sulfo-SMCC could be removed by washing several times. Next, the UCNPs and 2 nmol of SH-DNA were incubated in 2 mL of HEPES buffer solution and were shaken gently at room temperature overnight. The SH-DNA functionalized UCNPs were diluted with 2 mL of PBS (0.1 M, PH = 7.2) buffer solution after centrifugation and washing. The solution of SH-DNA functionalized UCNPs was stored at 4 °C for further use.

Preparation of mRNA detection device and detection of mRNA. 8 mg of EDC and 12 mg of NHS was dissolved in 1 mL of MES buffer to act as the activator, and then 2 μL of the activator was dropped to each PC dot and reacted for 10 min, followed by washing with ultrapure water. After drying, 2 μL of as-prepared mRNA probes was added to each PC dot and reacted at 30 °C for 30 min. Then, the PC dots were washed with PB buffer. For the

detection of mRNA, two kinds of experimental methods were carried out. In the first experimental method, the target mRNA was added first, and then the GO sheets were added.[4] In details, each PC dot was added 1 μL of targets containing TK1 mRNA and C-myc mRNA (TK1 perfectly matched target: 5'-AAGTATGCCAAAGACACTCGC-3', C-myc perfectly matched target: 5'-CCTCAACGTTAGCTTCACCAA-3'). After reacting at 25 °C for 2 h, the device was washed with PB buffer for one time. Then, 1 μL of GO (0.3 mg mL^{-1}) was dropped to each PC dot and reacted at 25 °C for 30 min to combine with the probes that without hybridization. Finally, the device was washed with ultrapure water for three times to remove the excess GO. A 980 nm CW laser and an 808 nm CW laser were used to irradiate the detection device and the luminescence images were captured by an unmodified camera phone. In the second experimental method, the GO sheets were added first to quench the luminescence of the mRNA probes, and then the target mRNA was added to restore the luminescence.[5, 6] In details, each PC dot was added 1 μL of GO (0.3 mg mL^{-1}) and reacted at 25 °C for 30 min to combine with the probes. Then, the PC dots were washed with ultrapure water for three times to remove excess GO. Finally, 1 μL of targets containing TK1 mRNA and C-myc mRNA was dropped to each PC dot and reacted at 25 °C for 2 h. The mRNA probes hybridized with the target mRNA, and GO sheets were released form the mRNA probes. The detection device was washed with ultrapure water for three times to remove the GO sheets released form the mRNA probes. As a result, extraordinary luminescence recovery was obtained after adding target mRNA. The same detection results were obtained with the use of two kinds of experimental methods, and the second experimental method was chosen to conduct the following experiment. Briefly, mRNA probes were immobilized on PC dots first, and then each PC dot was added 1 μL of GO (0.3 mg mL^{-1}) to bind with mRNA probes. Consequently, a flexible device for the detection of mRNA was constructed. The detection of two kinds of mRNAs with the above device was conducted by adding target mRNA to the mRNA detection device. A 980 nm CW laser and an 808 nm CW laser were used to irradiate

the detection device and the luminescence images were captured by an unmodified camera phone.

Cell culture. MCF-7 cell was cultured in RPMI-1640 medium with 0.01 mg mL^{-1} bovine insulin. The cell lines were supplemented with 10% fetel bovine serum and 100 U mL^{-1} antibiotics penicillin/streptomycin and maintained at 37 °C in a 100% humidified atmosphere containing 5% CO_2 at 37 °C.

mRNA extraction from MCF-7 cell or tissues. mRNAs were extracted using EasyPure® RNA Kit according to the manufacture's protocol. (1) Sample pretreatment: For cell, 10^5 MCF-7 cells were disrupted firstly by add 300–600 µL of Lysis/Binding Solution to cell plate and then the sample was centrifuged at 12,000 × g for 5 min and the supernatant of the sample was removed to the RNase-free tube. For tissue, 10 mg of tissue were frozen with liquid nitrogen at first, and then were grinded into powder. After that, the powder was removed to an RNase-free tube and 1 mL of Binding Buffer 4 and 15 µL of Proteinase K were added. After the blending processing, the mixture reacted at 56 °C for 15 min. The sample was centrifuged at 12,000 × g for 5 min and the supernatant of the sample was removed to the RNase-free tube. (2) Extraction of mRNA: 200 µL of 70% ethanol was added and mixed thoroughly. The sample was centrifuged at 12,000 × g for 30 sec and the supernatant was discarded. 500 µL of Cleaning Buffer 4 was added to the spin column and the sample was centrifuged at 12,000 × g for 30 sec and the supernatant was discarded. 500 µL of Washing Buffer 4 was added and the sample was centrifuged at 12,000 × g for 30 sec and the supernatant was discarded. Then, the spin column was centrifuged at 12,000 × g for 2 min to remove ethanol completely. The spin column was put to a new RNase-free tube and 100 µL of RNase-free water was added. The spin column was centrifuged at 12,000 × g for 2 min to elute mRNA to the tube.

Fresh samples from human breast cancer tissues and the corresponding non-tumor normal tissues were provided by Zhongnan Hospital (Wuhan University, China).

Detection of mRNA extraction from MCF-7 cell or tissues. 2 μL of mRNAs extracted from MCF-7 cells or tissues were added to the mRNA detection device and reacted at 25 °C for 2 h. Then, the detection device was washed with ultrapure water for three times to remove the GO sheets released form the mRNA probes. Finally, a 980 nm CW laser and an 808 nm CW laser were used to irradiate the detection device and the luminescence images were captured by an unmodified camera phone.

Luminescence image capture. A 980 nm CW laser and an 808 nm CW laser were used to irradiate the detection device. A mobile phone was used to capture the readout under different lighting conditions, using the camera's default autofocus and autoexposure settings.

References

[S1] F. Wang, R. R. Deng and X. G. Liu, *Nat. Protoc.*, 2014, **9**, 1634.

[S2] Y. Xiao, L. Y. Zeng, T. Xia, Z. J. Wu and Z. H. Liu, *Angew. Chem. Int. Ed.*, 2015, **54**, 5323.

[S3] M. V. Yezhelyev, A. Al-Hajj, C. Morris, A. I. Marcus, T. Liu, M. Lewis, C. Cohen, P. Zrazhevskiy, J. W. Simons, A. Rogatko, S. Nie, X. Gao and R. M. O'Regan, *Adv. Mater.*, 2007, **19**, 3146.

[S4] C. H. Liu, Z. Wang, H. X. Jia, Z. P. Li, *Chem. Commun.*, 2011, **47**, 4661.

[S5] Y. Wang, Z. H. Li, D. H. Hu, C. T. Lin, J. H. Li, Y. H. Lin, *J. Am. Chem. Soc.*, 2010, **132**, 9274.

[S6] C. H. Lu, H. H. Yang, C. L. Zhu, X. Chen, G. N. Chen, *Angew. Chem. Int. Ed.*, 2009, **48**, 4785.

Supplementary Figures

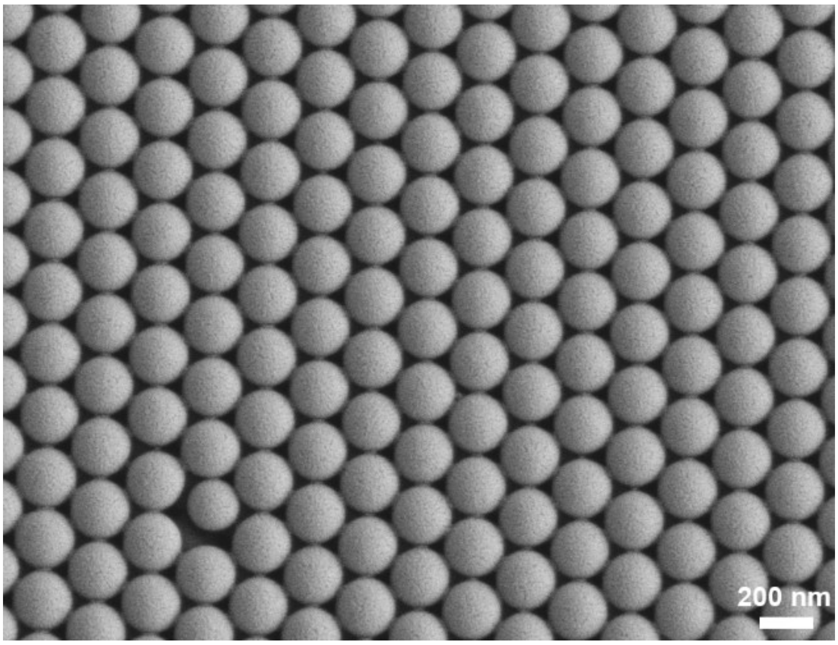

Fig. S1. SEM image of PC dot.

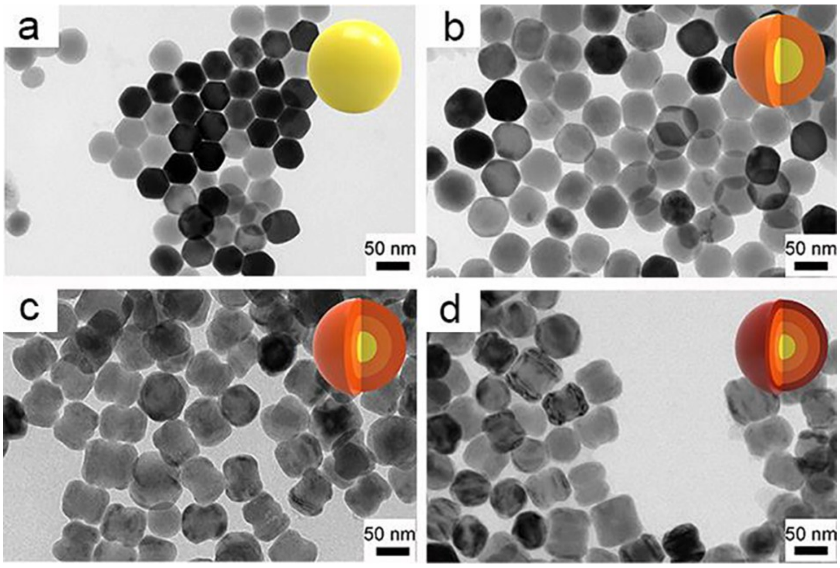

Fig. S2. TEM images of core-shell structured UCNPs. (a) TEM image of $NaYF_4$:Yb,Er. (b) TEM image of $NaYF_4$:Yb,Er@$NaYF_4$:Yb. (c) TEM image of $NaYF_4$:Yb,Er@$NaYF_4$:Yb @$NaNdF_4$:Yb. (d) TEM image of $NaYF_4$:Yb,Er@$NaYF_4$:Yb@$NaNdF_4$:Yb@$NaYF_4$:Yb. Inset: schematics of the core-shell structured UCNPs.

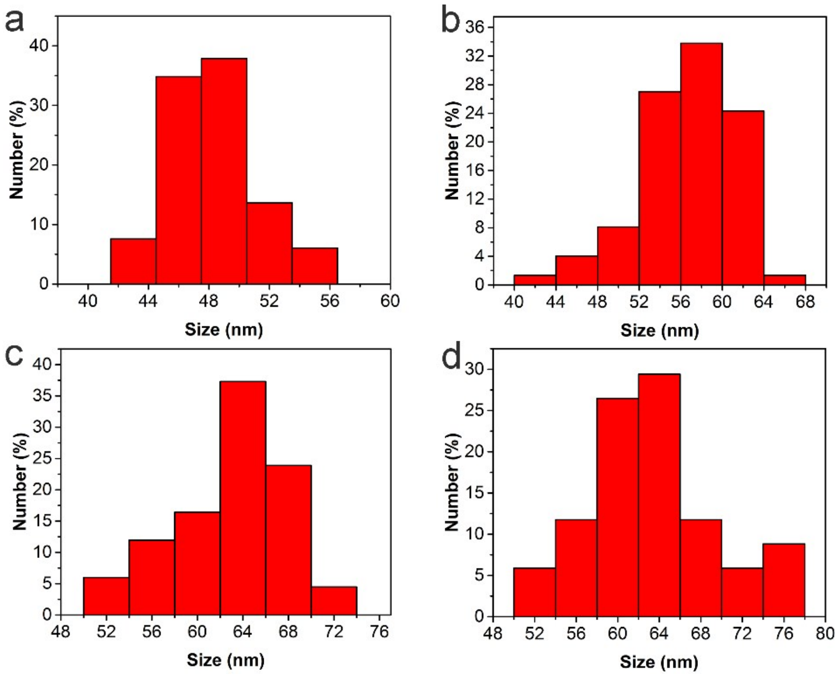

Fig. S3. The size distribution of $NaYF_4$:Yb,Er (a), $NaYF_4$:Yb,Er@$NaYF_4$:Yb (b), $NaYF_4$:Yb,Er@$NaYF_4$:Yb@$NaNdF_4$:Yb (c), and $NaYF_4$:Yb,Er@$NaYF_4$:Yb@$NaNdF_4$:Yb @$NaYF_4$:Yb (d).

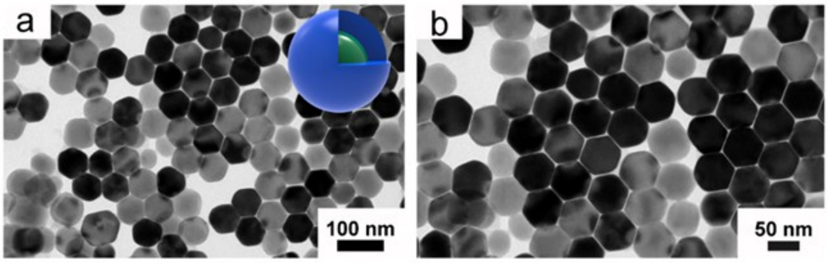

Fig. S4. TEM images of Tm-doped UCNPs at different magnifications.

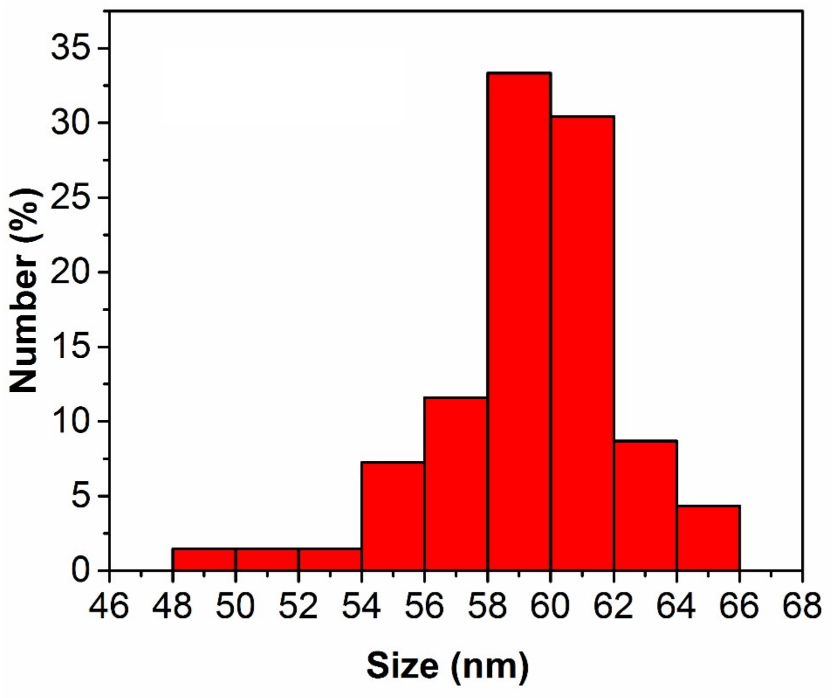

Fig. S5. The size distribution of the Tm-doped UCNPs.

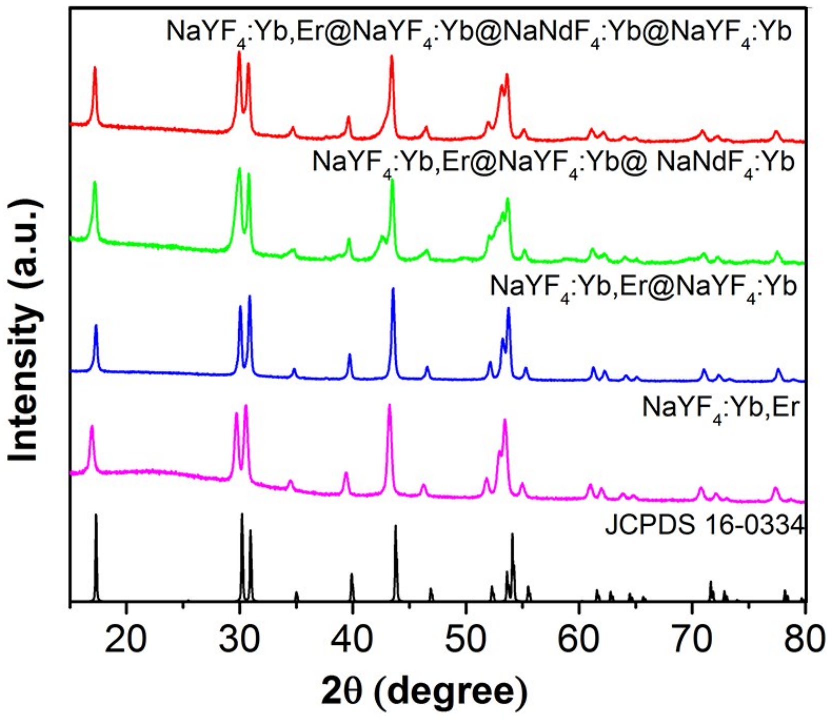

Fig. S6. X-ray powder diffraction (XRD) pattern of the Er-doped UCNPs.

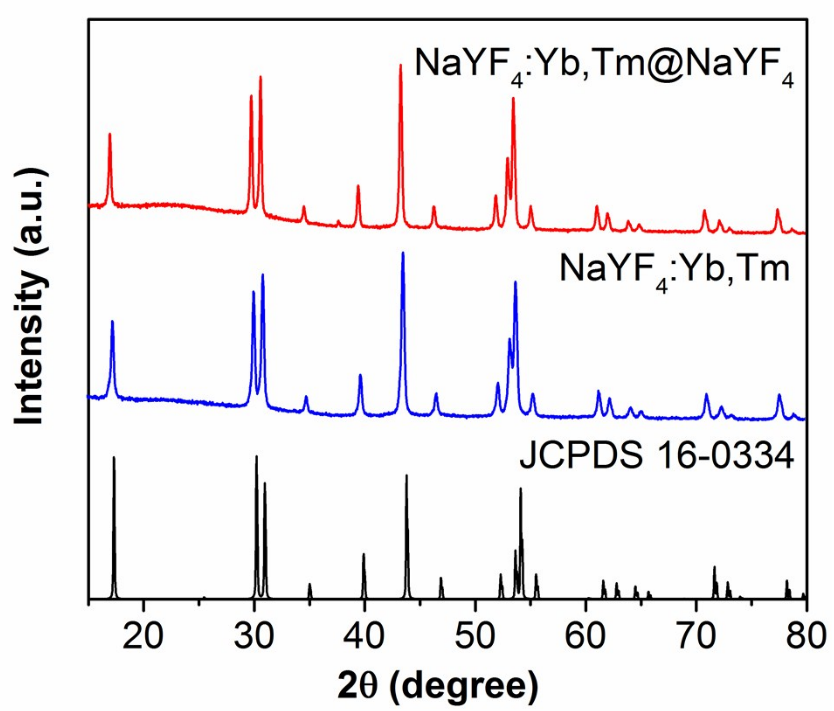

Fig. S7. XRD pattern of the Tm-doped UCNPs.

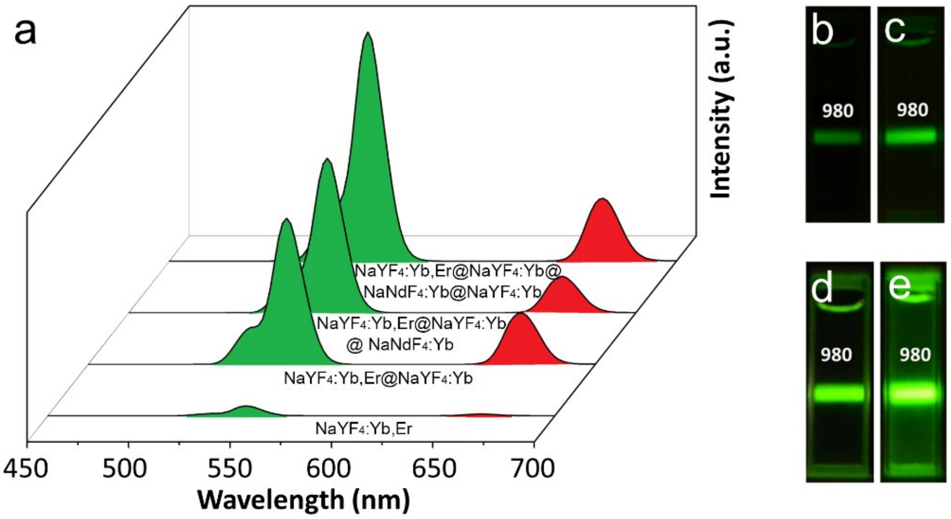

Fig. S8. (a) The UCL spectra of the core-shell structured UCNPs under the excitation of 980 nm. Luminescence photographs of $NaYF_4$:Yb,Er (b), $NaYF_4$:Yb,Er@$NaYF_4$:Yb (c), $NaYF_4$:Yb,Er@$NaYF_4$:Yb@$NaNdF_4$:Yb (d), and $NaYF_4$:Yb,Er@$NaYF_4$:Yb@$NaNdF_4$:Yb @$NaYF_4$:Yb (e) under CW 980 nm laser illumination, respectively.

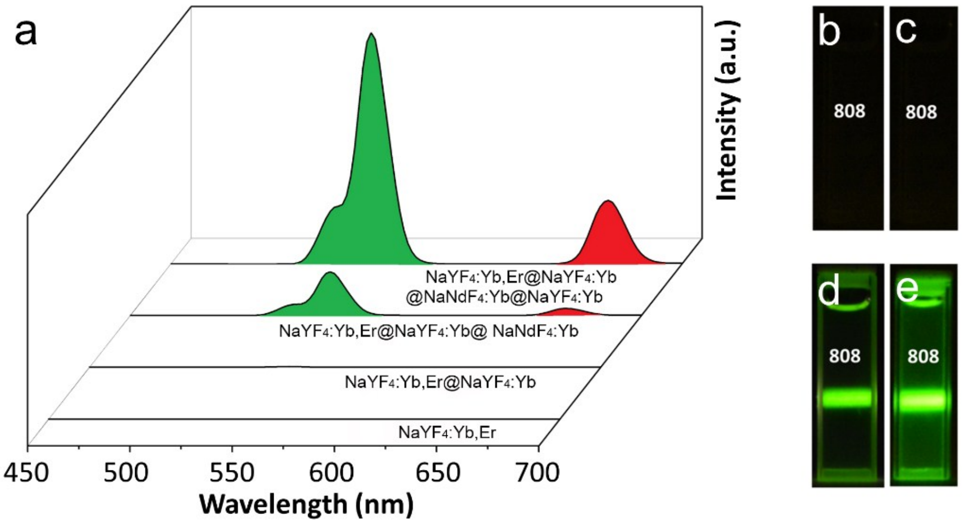

Fig. S9. (a) The UCL spectra of the core-shell structured UCNPs under the excitation of 808 nm. Luminescence photographs of $NaYF_4$:Yb,Er (b), $NaYF_4$:Yb,Er@$NaYF_4$:Yb (c), $NaYF_4$:Yb,Er@$NaYF_4$:Yb@$NaNdF_4$:Yb (d), and $NaYF_4$:Yb,Er@$NaYF_4$:Yb@$NaNdF_4$:Yb @$NaYF_4$:Yb (e) under CW 808 nm laser illumination, respectively.

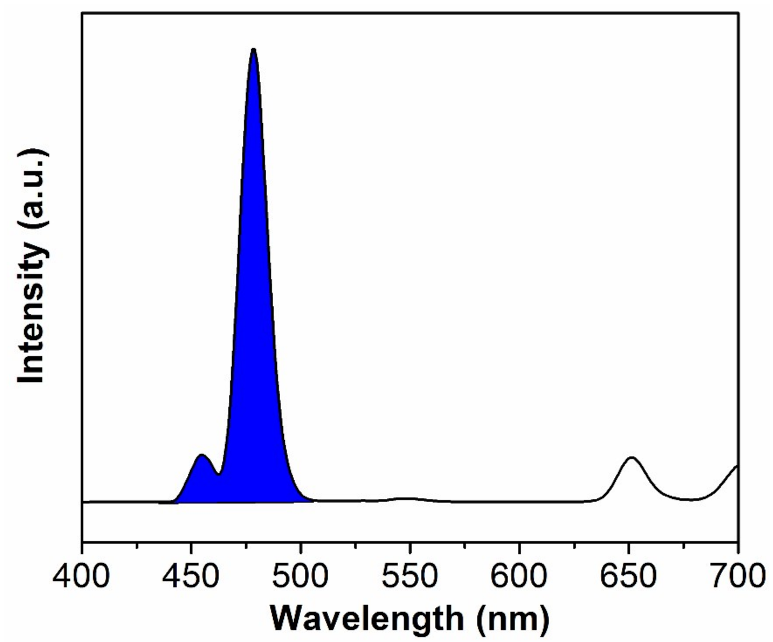

Fig. S10. The UCL spectrum of Tm-doped UCNPs under 980 nm excitation.

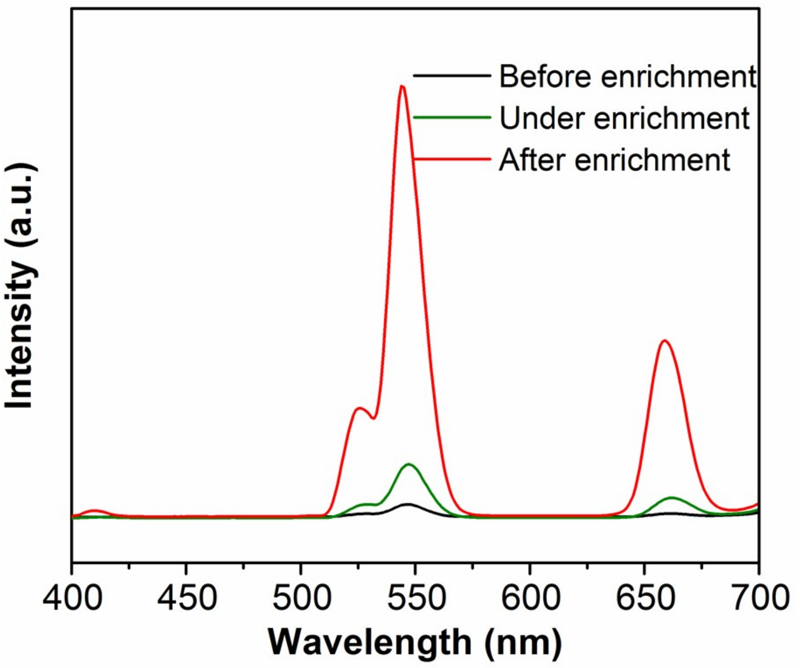

Fig. S11. The change of the luminescence intensity of Er-doped UCNPs under enrichment process.

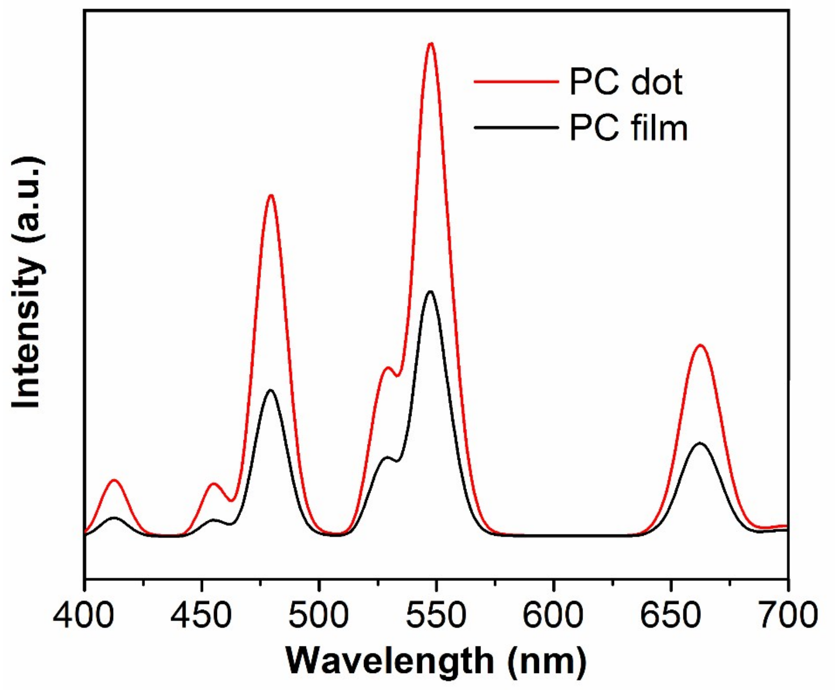

Fig. S12. The UCL intensity of Er-doped UCNPs on different substrates (black line: PC film, red line: PC dot).

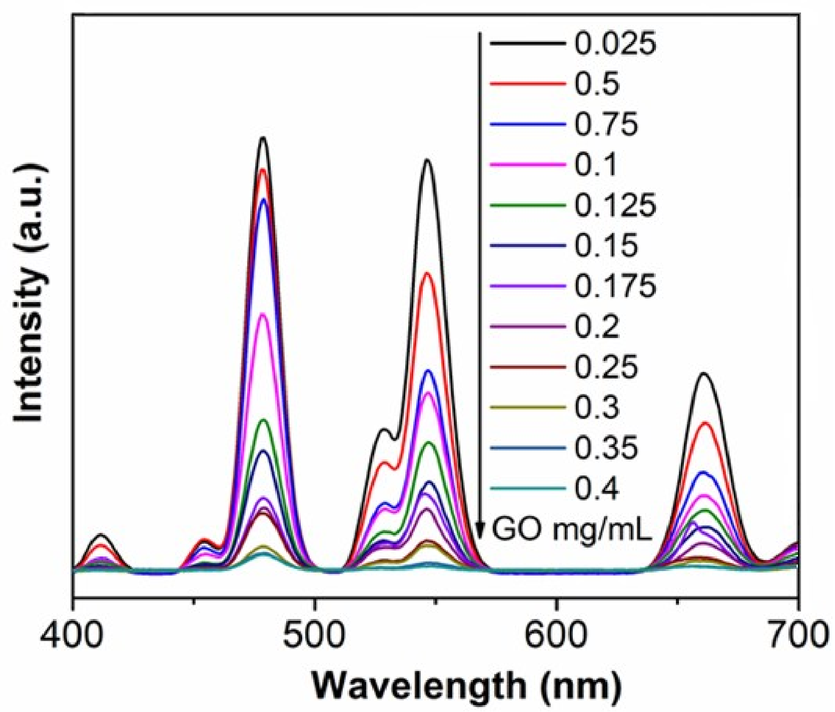

Fig. S13. Luminescence quenching of the UCNP-based probes with various concentrations of GO.

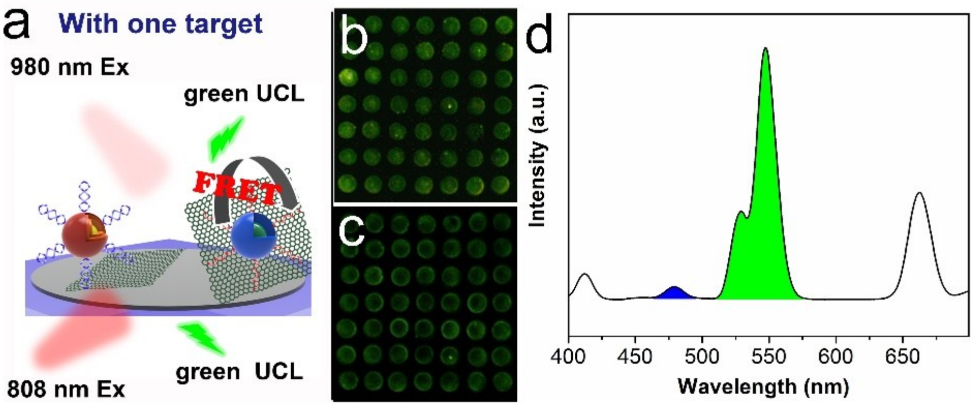

Fig. S14. (a) Working principle for the simultaneous detection of multiple mRNAs. The luminescence images of the mRNA detection devices in the presence of TK1 mRNA under 980 nm excitation (b) and under 808 nm excitation (c). (d) The UCL spectrum of the mRNA detection device with the addition of TK1 mRNA under 980 nm excitation.

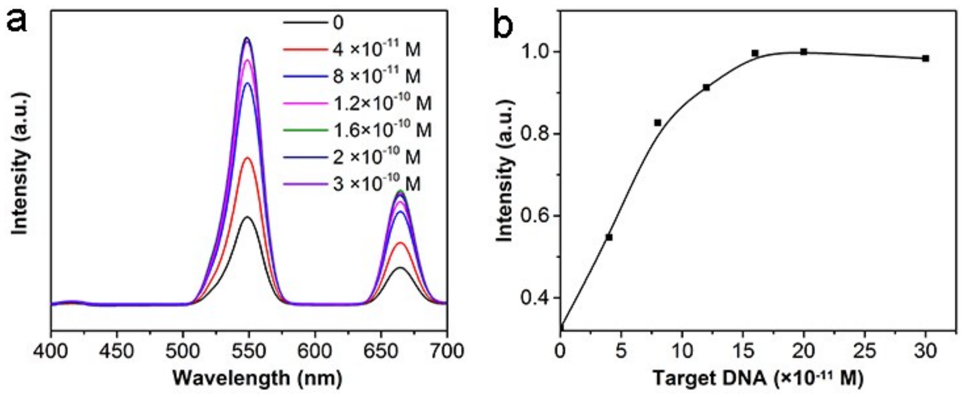

Fig. S15. (a) The UCL spectra of the mRNA detection devices with the addition of various concentrations of TK1 mRNA under 808 nm excitation. (b) The linear relationship between the UCL intensity and the concentration of TK1 mRNA.

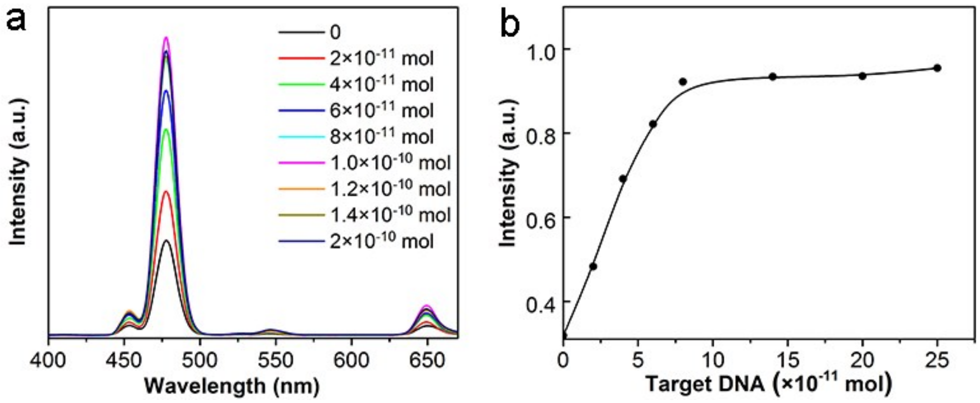

Fig. S16. (a) The UCL spectra of the mRNA detection devices with the addition of various concentrations of C-myc mRNA under 980 nm excitation. (b) The linear relationship between the UCL intensity and the concentration of C-myc mRNA.

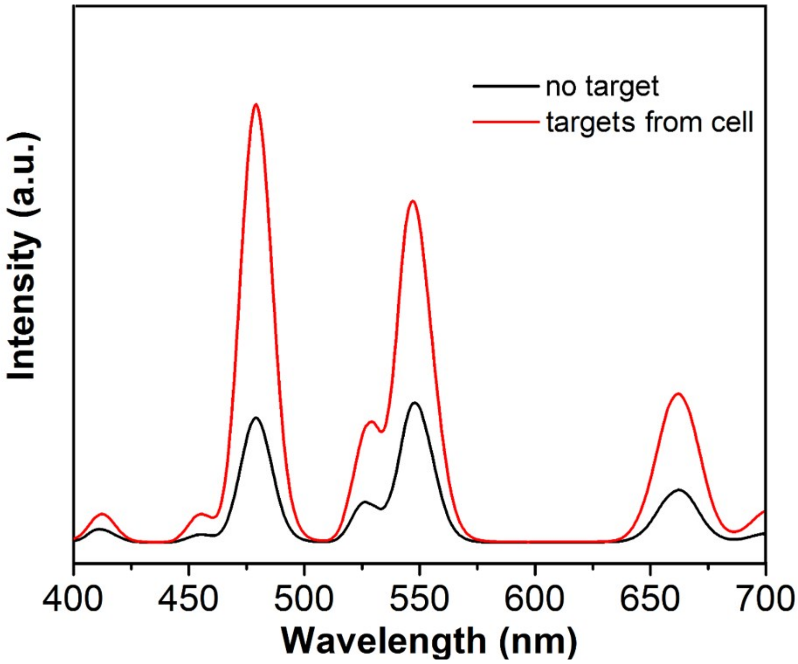

Fig. S17. The UCL spectra of the mRNA detection devices without the addition of target (black line) and with the addition of targets extracted from MCF-7 cell (red line) under 980 nm excitation.
